# Mnesic imbalance: a cognitive theory about autism spectrum disorders

**DOI:** 10.1186/1744-859X-7-20

**Published:** 2008-10-17

**Authors:** Miguel Ángel Romero-Munguía

**Affiliations:** 1 Hospital Psiquiátrico 'Dr. Samuel Ramírez Moreno', Autopista México-Puebla Km 5.5 Col. Santa Catarina, Del. Tláhuac, CP.13100, México City, México; 2 División de Estudios de Posgrado, Facultad de Medicina, Universidad Nacional Autónoma de México (UNAM), Ciudad Universitaria, México City, México

## Abstract

Autism is characterized by impairments in social interaction, communicative capacity and behavioral flexibility. Some cognitive theories can be useful for finding a relationship between these irregularities and the biological mechanisms that may give rise to this disorder. Among such theories are mentalizing deficit, weak central coherence and executive dysfunction, but none of them has been able to explain all three diagnostic symptoms of autism. These cognitive disorders may be related among themselves by faulty learning, since several research studies have shown that the brains of autistic individuals have abnormalities in the cerebellum, which plays a role in procedural learning. In keeping with this view, one may postulate the possibility that declarative memory replaces faulty procedural memory in some of its functions, which implies making conscious efforts in order to perform actions that are normally automatic. This may disturb cognitive development, resulting in autism symptoms. Furthermore, this mnesic imbalance is probably involved in all autism spectrum disorders. In the present work, this theory is expounded, including preliminary supporting evidence.

## Background

In 1943, Kanner described autism in 11 children lacking communicative language over a period of years [1]. He defined this disorder in his 1956 article: 'It is characterized by extreme aloneness and preoccupation with the preservation of sameness, and is manifest within the first 2 years of life' [2]. Asperger described children with similar symptoms, in addition to qualitatively abnormal communication and outstandingly original interests [3].

The American Psychiatric Association (APA) classifies autism as opposed to Asperger syndrome on the basis of a history of early speech delay in the former [4], but some authors refute this separation [5-7] and others view these disorders as autism spectrum disorders (ASD) [8,9]. regardless, the APA guides research on ASD through its Diagnostic and Statistical Manual of Mental Disorders (DSM) [4].

There is considerable evidence of neurobiological abnormalities in autism [10], and the need to explain how these abnormalities give rise to autism may justify the development of influential cognitive theories: theory of mind deficit, weak central coherence and executive dysfunction [11,12]. The theory of mind (mentalizing) is a system that enables one to infer thoughts, desires and other mental states. It may be used to explain and predict behavior of others [13]; Baron-Cohen *et al*. argued that a theory of mind deficit might explain social impairment in autism [14]. Central coherence is a tendency to create higher meanings from samples of data; Frith surmised that a weak central coherence might explain islets of ability and impaired social interaction of individuals with autism because 'they cannot see the forest for the trees' [15]. Executive function is a set of mental processes that help us control our actions. According to Russell and colleagues, executive dysfunction may explain the reduced behavioral flexibility of autistic individuals, who cannot understand alien actions because they cannot control their own [16].

These theories must meet universality (to be present in all autistic subjects), specificity (to be present only in autism) and precedence criteria (to be earlier than autism symptoms) [11,17], although this does not seem likely since there are autistic persons who pass mentalizing tasks [11]; if these tasks are more difficult, persons with normal development often perform worse than some individuals with autism [18,19]. In addition, autism symptoms are detected prior to the possibility of evaluating the theory of mind in healthy subjects [20,21]. Furthermore, there are autistic children with intact global processing of central coherence tasks [22,23]; it has been proposed that verbal difficulties are the critical factor for some deficits wrongly attributed to weak central coherence [24,25]. Executive dysfunction is present in several disorders and does not meet the universality criterion [12]. In addition, the executive function has not normally been developed during the period of infancy within which autism symptoms appear [16,20]. Obsessive desire for sameness, which Kanner considered a fundamental symptom of autism [1,2], is not explained by executive dysfunction [16], although this seems to be a better alternative than weak central coherence [12]; the theory of mind has same problem [11,16]. Under these circumstances, it has been suggested that these alterations may be related among themselves by an unidentified mechanism [11,16]; such a mechanism may be a mnesic imbalance, because if these cognitive disorders are acquired then development of these disorders could be due to learning alterations and learning's final product, the memory [26].

## Mnesic imbalance

Kanner pointed out the excellent rote memory of autistic children, as some autistic individuals can repeat sentences verbatim that they heard long ago. He even asked whether excessive information contributes to the development of autism [1]. By contrast, contemporary authors suggest a deficit of memory in autism [27-30]. This last proposition does not contradict the previous one, because each one is referring to a different type of memory. One is the declarative memory, which allows us to consciously remember facts and events, while the other is the procedural memory, which allows one to carry out actions automatically [31,32].

Suggesting that autistic children store information without the abstraction required for its use in verbal communication, Hermelin and O'Connor considered the possibility of a deficient abstract memory [33]. Also, Goldberg surmised that hyperlexic children or with other savant syndromes have dysfunctional procedural memory, though their declarative memory is relatively intact. All this might be reflected in restricted behaviors and in the inability to manipulate their knowledge [27]. Moreover, Gustafsson believes that procedural memory normally consists of essential features, but that it consists of salient and unimportant details in autistic individuals; for instance, the color of the walls in any bathroom [34]. However, this proposal is not in accordance with the implicit nature of procedural memory [26,31]. By contrast, an imbalance between procedural and declarative memories may explain autism symptoms according to the nature of memory, even in children without islets of ability [28,30]. This last proposition is consistent with data from several studies [29,30,35]. In one of them, utilizing the Serial Response Time Task (SRTT), a procedural learning task, the data suggest that in individuals with high functioning autism acquisition of procedural knowledge is impaired [29]. In another study, evaluating the ability to recognize words from a target list among the items of a recognition test, adults with ASD had a more accurate declarative memory than normal individuals [35].

In order to investigate the possible implication of a faulty procedural memory in the psychopathology of infantile autism, declarative memory (lexicon) and procedural memory (gestural responses) were studied in autistic children and control patients with developmental mixed receptive-expressive language disorder. In both groups, receptive language was significantly below that expected for their age, but the lack of procedural memory and the positive correlation between autism symptoms and declarative memory achieves statistical significance only in the sample of children with autism, which suggests an imbalance between declarative and procedural memory in autism rather than mere faulty procedural memory [30], this interpretation is in agreement with the declarative/procedural model, which assumes that mental lexicon depends on declarative memory [36]. Furthermore, the automatic nature of the responses from autistic children to instrumental gestures made by others indicates that the gestural responses are a measure of procedural memory [15,26,31]. Another study has reported a similar result, observing significantly higher scores on eight items and one subscale of the Autism Behavior Checklist in the autism-verbal group than in the autism-mute group, although the difference between groups at the full scale did not reach statistical significance in that study [37].

## Genesis of diagnostic symptoms

According to the above a mnesic imbalance is probably involved in the genesis of the three diagnostic symptoms of autism, but the question remains: how does the mnesic imbalance give rise to autism symptoms? Two Spanish-language manuscripts have supplied possible answers [28,30], but new information has subsequently appeared. The present work aims to update and review this theory; for this reason, the diagnostic criteria for ASD will be mentioned in order of convenience for explaining the present theory, rather than the order in which they appear in the third and fourth revised editions of the DSM [4,38].

### Impairment in behavioral flexibility

Procedural memory enables us to carry out activities without giving them conscious thought [39], so a lack of procedural memory may increase our need to pay attention to our own hands, feet and objects [40]; that is, the need to look, touch, smell and suck. However, autistic children can improve their procedural learning if they perform activities, the results of which are foreseeable and immediate: manipulating taps, spinning wheels of toy cars, or controlling lamps [41,42]. All these actions are called persistent preoccupation with parts of objects (DSM criterion) [4,38].

The less variability among the qualities of the objects result in an easier initial procedural learning [43], which also is achieved by environmental sameness [32]; therefore, the marked distress over changes in trivial aspects of the environment (DSM criterion) [38], and the obsessive desire for sameness [2] may be justified.

A deficit of procedural learning complicates the development of automatic actions [26,31], which apparently occurs during the first months of life in subjects with ASD [44,45]. Under these circumstances, they may react as typically-developing infants in a position to develop a calibration of movement directionality: looking at their limbs significantly longer as well as moving them more vigorously in order to increase procedural memory and self-knowledge [26,46]. Perhaps autistic children get the same result when increasing proprioceptive and tactile stimulation during swimming [47], using cylindrical hinged elbow splints [48], or controlling a self-immobilizing machine [49]; therefore, rocking, swinging, spinning, flapping, finger flicking, tiptoe walking and jumping (DSM criterion) [4,38,40,41], might serve this purpose.

Using procedural learning, it is possible to develop postural control without perceiving our own sway, which plays an important role in the maintenance of posture [50]; moreover, persons with ASD suffer underdevelopment of postural control [51,52]. Whereas persons developing normally have a 'frozen sway' so that the declarative memory can be free to focus on another task [53], some persons with ASD have a outstanding sway [40,41], and they may be using their conscious thought to avoid falling.

A sequence of actions that does not vary is more suitable than several sequences for facilitating procedural learning [42,43]. This and the other strategies already described may explain the insistence on following routines in precise detail (DSM criterion) [4,38], but these strategies are not suitable for major development of procedural memory [32,42,43]; thus, they would decrease together with repetitive behaviors, although not all of them. Indeed, circumscribed interests are increased in high-functioning individuals with ASD [41,54,55], which may be explained by the contribution of declarative memory to circumscribed interests. However, the increase in episodic memory is smaller than the increase in semantic memory in persons with ASD [56], since the contribution from procedural knowledge to retrieval of episodic memory may be faulty [57]. This may explain why retrieval of sequences of actions to be executed is difficult for some high-functioning individuals with ASD [49], why they prefer reading a book (static elements) than watching TV (dynamic elements) or why they prefer either to playing video games (actions) [54], and why some autistic adults prefer playing an instrument or memorizing encyclopedic amounts of information although they have not yet learned to dress themselves nor tie their own shoes [9,58]. Perhaps all this allows them to show their abilities instead of their disabilities caused by mnesic imbalance [30,47,56]. This may also explain the patterns of interest that are abnormal either in intensity or focus (DSM criterion) [4,38].

### Impairment in communicative capacity

Procedural memory seems important for learning and categorizing of phonemes [59,60], so a lack of procedural memory may lead to mutism, few phonemes or reduced vocabulary [61]. However, many people with ASD would be able to sacrifice some acoustic features of the phonemes, such as amplitude variation and pitch level, in order to encompass all the phonetic categories of the native language, albeit with atypical features [62], producing a peculiar voice and prosodic deficits [40,61]; nonetheless, repetition may significantly improve procedural memory [26], which may convert some individuals with ASD into good imitators of prosody [63], who simply repeat what others said (echolaly) [40,41], what they themselves said (palilaly) [64], or phrases that may seem irrelevant to the present situation [1]. However, the speech thus acquired is not useful for communication because the meaning of words employed may be unknown to them [65] due to perceptual categorization that may require procedural learning [66]. This may be the case with, for example, the categories of fruit and mental state expressed in eyes [18,67], but even an autistic person with knowledge of categories would not be able to understand the meaning of sentences [30,68] because the brain network associated with procedural learning is activated during sentence comprehension, likely, in order to 'reconstruct' in the brain the actions described by others [39,50,69]. Indeed, normally-developing children may use mental simulation to place themselves as the protagonist of a narration at the time they are selecting a deictic term, despite a contrary declarative knowledge [70]; the same 'rebelliousness' is manifest when they say 'breaked' instead of 'broke' [71], so that procedural memory is used instead of declarative memory, resulting in inductive logic answers [72]. All of this is in accordance to the declarative/procedural model, which assumes that morphology and syntax depend on procedural memory [30,36]. Faulty procedural learning may complicate the simultaneous application of the elements of verbal communication, for example the verbal intonation of children with ASD is better in repetition tasks (declarative memory) than in spontaneous speech (procedural memory) [73]. This lack of simultaneousness also explains repetitive non-communicative speech; the difficulty in inferring appropriate words such as prepositions, adjectives, adverbs and deictic terms for the person (I, you, he, she, my, your, etc.), things (this, that, these, those), places (here, there, above, below, etc.), and times (now, tomorrow, yesterday); finally, sentences with grammar divorced from their context [4,17]. All these are considered marked abnormalities in the production, form and content of speech (DSM criteria) [4,38].

Procedural learning is important to acquire phonemes and meanings [60,66]; this is why ironic speech may involve procedural memory [74]. Indeed, the pragmatic difficulties in ASD seem to arise from inference deficits caused by faulty procedural memory [72,75]. Consequently, some persons with ASD initiate their 'conversations', with frequent irrelevant remarks, that are nonetheless very well practiced because they do not want to appear as fools; however, the failure of this strategy is obvious from the marked impairment in the ability to initiate or sustain a conversation with others (DSM criterion) [4,38,47].

The absence of imaginative activity (DSM criterion) [38] might also occur because of the problems surrounding the learning of concepts and categories [66], and inductive reasoning [75,76]. In addition, the absence of real objects in simulation games may be a setback to faulty procedural learning, though that is an advantage for preserved procedural learning [32,42,77].

Whereas the ability for inductive reasoning in children with ASD is poor, their deductive reasoning ability is good [75,76], but their deductive reasoning ability may appear poor if the correct answers are inconsistent with the facts and it is difficult to disentangle what is more important [78]. This means that fantasy can convert a deductive problem into an inductive problem, perhaps explaining the lack of interest among children with ASD in imaginative activities (DSM criterion) [38].

Some autistic persons can neither verbally communicate nor use gestural communication (DSM criterion) [4,38], even if they know the meaning of many words [30,68]. This may be because automatic instrumental gestures perhaps depend on procedural memory [15,26,50], while lexicon depends on declarative memory [36]. Indeed, automatic mimicry is impaired in autistic persons, whereas their voluntary mimicry is maintained [79], which explains why autistic people who have a good level of verbal comprehension have no trouble with instrumental gestures but do have trouble with expressive gestures [15], in other words, trouble with gestures to regulate social interaction (DSM criterion) due to instrumental gestures are easily translated to verbal language, while expressive gestures are not [18,80,81]. Additionally, autistic people who have a low level of verbal comprehension have trouble with imitation (DSM criterion) [38,40]. This would be related to the so-called 'mirror neurons' in children with ASD [82], since the familiar elements (procedural knowledge) of the observed novel movements might generate resonant activity within the mirror neuron system and thus facilitate procedural learning [83].

### Impairment in social interaction

It has been proposed that gestural and verbal languages are essential for socialization and are not only instruments [84], so the lack of social or emotional reciprocity, the abnormal seeking of comfort, the lack of spontaneous seeking to share interests and the abnormal social play (DSM criteria) [4,38] may be viewed as result of impaired language. By contrast, these symptoms would be defined as impairments in dyadic orienting, joint attention and response to requesting [85].

The failure to develop peer relationships appropriate to developmental level (DSM criterion) [4] implies that autistic individuals are better at understanding physical systems than at understanding the minds of people. This is consistent with the empathizing/systemizing model, which proposes that systemizing works for deterministic phenomena with an exact explanation, whereas empathizing involves an imaginative leap in the absence of much data, whose causal explanation is at best a 'maybe' [86]. It has been suggested that systemizing in individuals with ASD is good and their empathizing is poor [19]; however, their systemizing may exhibit poor performance if systemizing problems are formulated as inductive problems [76], whereas their empathizing may improve significantly if the empathizing problems are formulated as deductive problems using explicit representations [87]; consequently, some persons with ASD may be able to acquire an explicit theory of mind [5]. Indeed, some persons with ASD use explicit mental representations to resolve systemizing problems, while normally developing individuals do not [49,88].

## Conclusion

The mnesic imbalance theory proposes that all three diagnostic symptoms of autism may be explained by cognitive disorders due to the mnesic imbalance between a faulty procedural memory and a relatively preserved declarative memory; in other words, the majority of autism symptoms may be viewed as attempts to compensate for deficits in procedural learning [28,30], while the sensory disturbances and other symptoms [38] may be directly explained by faulty procedural memory [59,60].

Also, cerebellar maldevelopment may cause faulty procedural memory and brain overgrowth may be associated to a greater use of declarative memory; both are the most repeated findings in autism neuroanatomy [10]. However, additional empirical studies are needed.

## List of abbreviations

APA: American Psychiatric Association; ASD: autism spectrum disorders; DSM: Diagnostic and Statistical Manual of Mental Disorders.

## Competing interests

The author declares that they have no competing interests.

## Authors' contributions

MÁRM is the sole contributor to this review.
